# Mucosal-Associated Invariant T Cells in the Digestive System: Defender or Destroyer?

**DOI:** 10.1016/j.jcmgh.2022.12.014

**Published:** 2022-12-27

**Authors:** Hejiao Zhang, Haiyuan Shen, Liangliang Zhou, Linxi Xie, Derun Kong, Hua Wang

**Affiliations:** 1 Department of Gastroenterology, the First Affiliated Hospital of Anhui Medical University, Hefei, China; 2 Department of Oncology, the First Affiliated Hospital of Anhui Medical University, Hefei, China; 3 Inflammation and Immune Mediated Diseases Laboratory of Anhui Province, Anhui Medical University, Hefei, China; 4 School of Basic Medical Science, Anhui Medical University, Hefei, China

**Keywords:** MAIT Cells, MR1, Digestive Diseases, Cytokines, Clinical Therapy, ALD, alcohol-related liver disease, BE, Barrett's esophagus, EAC, esophageal adenocarcinoma, HCC, hepatocellular carcinoma, HCV, hepatitis C virus, IBD, inflammatory bowel diseases, IFN, interferon, IL, interleukin, MAIT, mucosal-associated invariant T, MR1, major histocompatibility complex class I–related protein, NAFLD, nonalcoholic fatty liver disease, PBC, primary biliary cholangitis, T-bet, T-box expressed in T cell, TCR, T-cell receptor, TNF, tumor necrosis factor

## Abstract

Mucosal-associated invariant T (MAIT) cells are a subset of innate T lymphocytes that express the semi-invariant T cell receptor and recognize riboflavin metabolites via the major histocompatibility complex class I–related protein. Given the abundance of MAIT cells in the human body, their role in human diseases has been increasingly studied in recent years. MAIT cells may serve as targets for clinical therapy. Specifically, this review discusses how MAIT cells are altered in gastric, esophageal, intestinal, and hepatobiliary diseases and describes their protective or pathogenic roles. A greater understanding of MAIT cells will provide a more favorable therapeutic approach for digestive diseases in the clinical field.

**Summary**

This review describes the role played by mucosal-associated invariant T cells in gastric, esophageal, intestinal, and hepatobiliary diseases and discusses the prospects for the use of the cells in clinical therapy.

The digestive system is one of the most significant systems of the human body and involves the digestive barrier, which protects the mucosa from harmful substances from the digestive tract, such as bacteria and toxins.^1^ This barrier is typically maintained by immune cells, such as macrophages, natural killer cells, and T cells. As a very abundant class of innate T cells in the human body, mucosal-associated invariant T (MAIT) cells are expected to play a very important role in human diseases.^2^ It is estimated that almost all digestive organs contain MAIT cells, with the intestine and liver having the largest number of MAIT cells in the human body.^3^ MAIT cells have been studied in various inflammatory and neoplastic diseases of the digestive system. A variety of cytokines and cytotoxic molecules are produced by MAIT cells and induce different effects on different diseases. Throughout this article we describe the characteristics of MAIT cells and their role in human and mouse digestive system diseases.

## Characteristics, Distribution, and Activation of Mucosal-Associated Invariant T Cells

### Characteristics of Mucosal-Associated Invariant T Cells

MAIT cells are a subset of innate unconventional T lymphocytes expressing the semi-invariant T-cell receptor (TCR) and are involved in immunomodulatory effects.^4^^,^^5^ In humans, MAIT cells express TCR Vα7.2-Ja33 paired with a limited array of TCR β-chains. In mice, MAIT cells express TCR Vα19-Ja33, paired with Vβ6 or Vβ8.^6^^,^^7^ MAIT cells expressed activation markers, such as CD69, CD25, and CD38; degranulation marker CD107; exhaustion markers, such as PD-1 and CTLA-4; and transcription factors, such as promyelocytic leukemia zinc finger, T-box expressed in T cell (T-bet), and RAR-related orphan receptor γt.^8^, ^9^, ^10^ Mature MAIT cells in mice were divided into 2 subsets based on the expression and distribution of T-bet and RAR-related orphan receptor γt: MAIT1 and MAIT17. MAIT1 cells expressing T-bet secreted interferon (IFN)-γ and were mainly located in the liver and spleen. MAIT17 cells expressing RAR-related orphan receptor γt–secreted interleukin (IL)-17A and were mainly located in mucosal tissues, such as lung, skin, and intestine. In contrast, human MAIT cells do not have MAIT1 and MAIT17 subsets.^10^, ^11^, ^12^ Based on the expression of CD4 and CD8 coreceptors, human circulating MAIT cells are mainly classified into CD8^+^ and CD4^-^CD8^-^(DN) subsets, and CD4^+^ MAIT cells are observed at a very low frequency. However, most mouse MAIT cells mainly belong to the CD4^-^CD8^-^ (DN) subset.^4^^,^^13^ Peripheral blood MAIT cells express different combinations of chemokine receptors that mediate the migration of MAIT cells to peripheral tissues, including the gut-homing chemokine receptors α4β7 and CCR9, and the receptors CCR6 and CXCR6 involved in hepatic homing.^14^, ^15^, ^16^, ^17^ CD161 is often used in conjunction with Va7.2 as a marker for MAIT cells.^18^ However, CD161 is expressed by non-MAIT cells in human tissues. Some of the studies using this marker may thus have misidentified MAIT cells in nonlymphoid tissues.^17^ Reantragoon et al^6^ found that the MR1-5-OP-RU tetramer could be used to identify MAIT cells and defined the phenotypic characteristics of human and mouse MAIT cells in 2013.

### Distribution of Mucosal-Associated Invariant T Cells

MAIT cells are widely distributed in the human body and are located in the liver, intestine, lungs, blood, skin, and female reproductive mucosa.^6^^,^^11^, ^12^, ^13^^,^^19^, ^20^, ^21^, ^22^, ^23^, ^24^, ^25^ Nonhuman primates do not have as many MAIT cells as humans, but their distribution in the liver and mucosal tissues is similar to that of humans.^26^ The frequency of MAIT cells in commonly used laboratory mice (C57BL/6J and BALB/c) is much lower than in humans.^11^^,^^12^^,^^21^ However, the frequency of MAIT cells was 20-fold higher in wild mice captured in Thailand than C57BL/6J mice.^27^ Researchers frequently use MAIT cell–deficient mice (MR1^-^/^-^) and MAIT cell–rich mice (Vα19TCRTg) to explore the role of MAIT cells in disease.^28^, ^29^, ^30^ Different age groups have different MAIT cell frequencies; these cells increase gradually from newborns to adult humans, reaching their peak in adulthood and then gradually declining.^31^^,^^32^

### Activation of Mucosal-Associated Invariant T Cells

**Figure 1:**
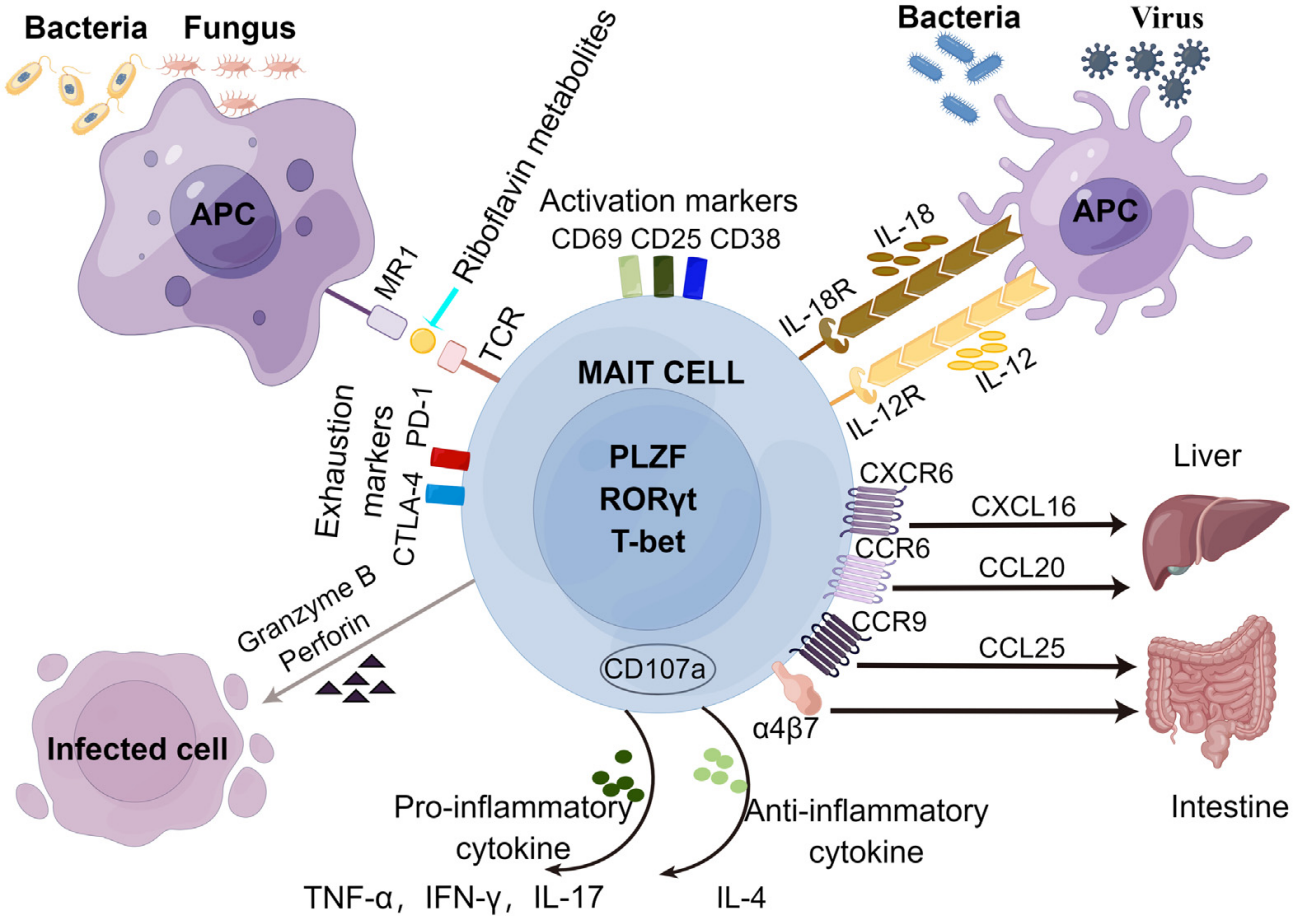
**Phenotype, function, and activation pathways of MAIT cells.** MAIT cells secrete cytotoxic molecules, including perforin and granzyme B; proinflammatory cytokines, such as TNF-α, IFN-γ, and IL-17; or anti-inflammatory cytokine, such as IL-4; and express a series of activation and exhaustion markers, transcription factors, and homing factor receptors on stimulation of their TCR by riboflavin-derived ligands bound to MR1 or in response to cytokines. α4β7, integrin α4β7; APC, antigen-presenting cells; CCR, CC-chemokine receptor; CXCR, CXC-chemokine receptor; PLZF, promyelocytic leukemia zinc finger; RORγt, RAR-related orphan receptor γt.

In humans and mice, MAIT cells can be activated by 2 pathways (Figure 1). First, MAIT cells are activated on binding of the major histocompatibility complex class I-related protein (MR1) of antigen-presenting cells to metabolites of the bacterial riboflavin pathway, which is also known as the MR1-dependent pathway.^2^^,^^33^, ^34^, ^35^ This pathway can be activated during infections by most bacteria and fungi that can synthesize riboflavin,^36^^,^^37^ such as *Helicobacter pylori*,^38^ *Legionella longbeachae*,^39^ *Escherichia coli*,^36^ and *Streptococcus pneumoniae*.^40^ MAIT cells rapidly produce a variety of cytokines, including IFN-γ, tumor necrosis factor (TNF)-α, IL-17, and IL-22.^36^^,^^41^ Second, MAIT cells are activated by the MR1-independent (cytokine) pathway. Indeed, these cells express receptors for cytokines, such as IL-12 and IL-18, which can trigger the secretion of perforin and granzyme B,^42^^,^^43^ which exhibit cytotoxic functions. This pathway can be activated during infections by viruses and a few bacteria, such as hepatitis C virus (HCV), dengue virus, and *Enterococcus faecalis*, and so forth.^44^^,^^45^ Occasionally both pathways act together to enhance the stimulatory effect; for example, IL-7 enhances cytokine production by MAIT cells dependent on MR1.^3^ The respective role of either pathway is commonly determined by adding anti-MR1 antibodies or cytokine neutralizers to experiments (Figure 1).^44^^,^^46^^,^^47^

## Mucosal-Associated Invariant T Cells in Gastric and Esophageal Diseases

### Gastritis

More than 50% of the world's population has been infected with *H pylori*, and approximately 80% or more of the populations of individual countries has been infected with the bacteria.^48^, ^49^, ^50^ This bacterial infection initially causes chronic gastritis, which may also develop into gastric ulcers and ultimately gastric cancer.^51^ The presence of MAIT cells in the stomach was first identified by Booth et al.^25^ Patients with *H pylori*–infected gastritis had lower peripheral blood MAIT cells than uninfected patients but increased production of proinflammatory cytokines, such as TNF-α, IFN-γ, IL-17, and IL-9. It was also found that MAIT TCR transgenic mice infected with *H pylori* showed severe gastric atrophy, indicating that MAIT cells play a pathogenic role in this disease.^38^^,^^52^

### Gastric and Esophageal Cancers

Gastric and esophageal cancers are common tumors of the upper gastrointestinal tract. In patients with gastric cancer, the frequency of peripheral blood MAIT cells and serum granzyme B levels were significantly lower before and after chemotherapy compared with healthy volunteers, and the changes of MAIT cells in gastric tissues and the role of MAIT cells in gastric cancer awaits further demonstration.^53^ Not only are MAIT cells present in gastric mucosa, but Melo et al^54^ found that MAIT cells are also present in human esophageal tissue, and including in Barrett's esophagus (BE), a precancerous lesion of esophageal adenocarcinoma (EAC), which increases the risk of EAC development more than 10-fold. BE and EAC showed reduced peripheral blood MAIT cell frequencies compared with healthy control subjects, whereas BE tissue and EAC tumor tissue contained higher densities of MAIT cells than adjacent normal tissue. It is possible that altered flora in BE and EAC tissues or migration of MAIT cells from peripheral blood resulted in increased levels of MAIT cells in tissues. Interestingly, the viability of OE33 cells (human EAC cell line) was reduced when expanded MAIT cells were cocultured with them in vitro, and EAC patients with high MAIT cell frequency in tumor tissues had a better prognosis, overall suggesting an antitumor effect of MAIT cells in EAC.^54^

## Mucosal-Associated Invariant T Cells in Intestinal Diseases

### Inflammatory Bowel Diseases

Inflammatory bowel diseases (IBD) are commonly divided into ulcerative colitis and Crohn's disease.^55^ A reduced frequency of circulating MAIT cells was found in patients with IBD, and the decrease correlated with disease severity.^16^^,^^56^, ^57^, ^58^, ^59^ In contrast, the number of MAIT cells was higher in inflamed intestinal tissue than in autologous noninflamed tissue.^56^, ^57^, ^58^, ^59^ Expression of CD69, PD-1, and annexin V was increased in circulating MAIT cells, whereas expression of such cytokines as CCL20, CXCL10, CXCL16, and CCL25 was enhanced in inflamed intestinal tissues. It was therefore suggested that the decrease in peripheral MAIT cells may be caused by activation-induced cell death or MAIT cell migration to tissues.^16^^,^^56^^,^^57^ A distinct result was obtained by Hiejima et al,^16^ who reported a lower number of inflamed intestinal MAIT cells in patients with IBD compared with control subjects possibly because inflamed and noninflamed intestinal tissues were compared between different individuals.^16^ In patients with IBD, MAIT cells produced less IFN-γ and more IL-22, suggesting a possible anti-inflammatory role of MAIT cells in IBD.^57^^,^^58^ However, Serriari et al^58^ found increased IL-17 secretion by circulating MAIT cells and therefore speculated that MAIT cells may play a pathogenic role in IBD. Yasutomi et al^30^ further used oxazolone to induce colitis in mice and found milder inflammation in MR1^-^/^-^ mice than control animals, indicating that MAIT cells are detrimental in this mouse model of intestinal inflammation.

### Colorectal Cancer

**Figure 2:**
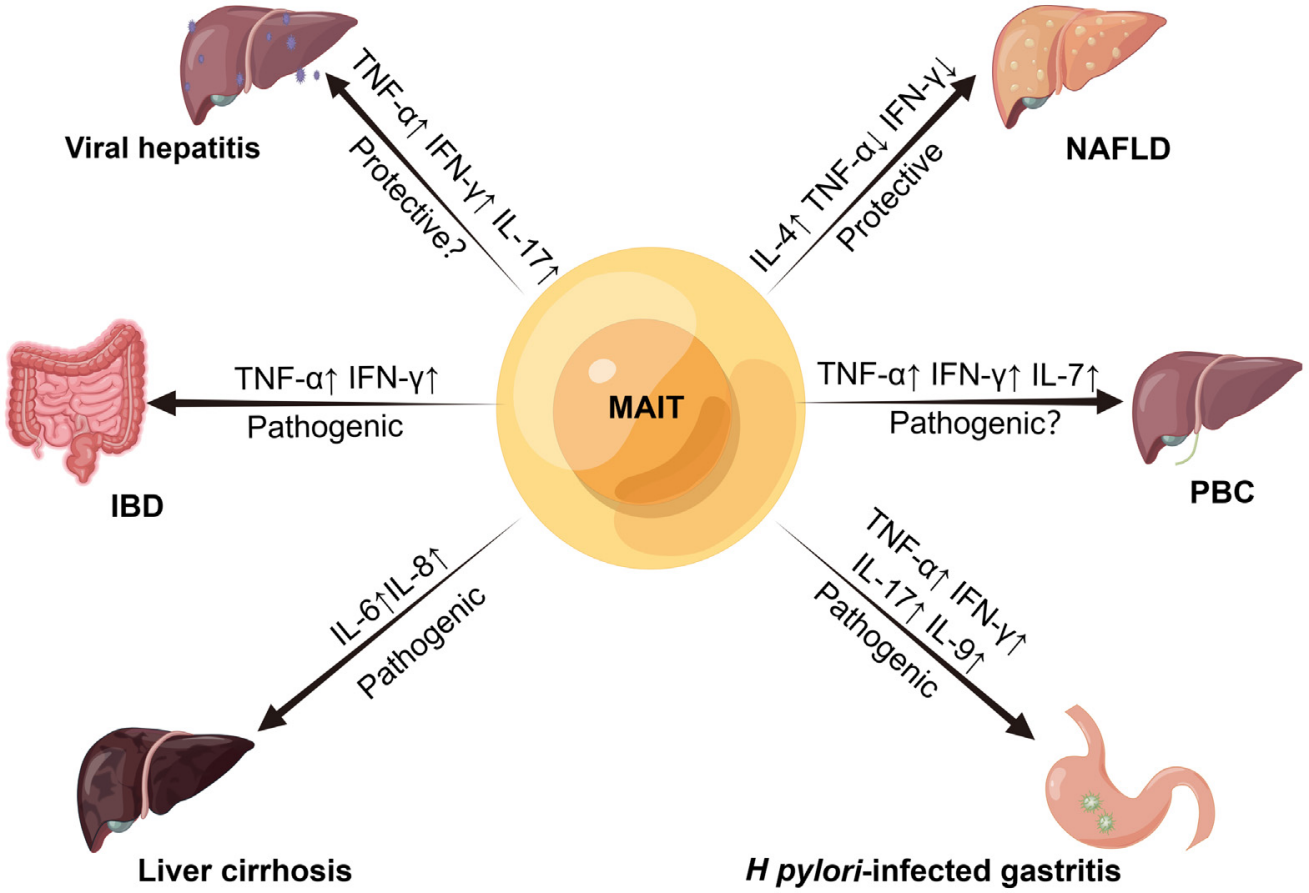
**Role of MAIT cells in hepatic and gastrointestinal diseases.** Activated MAIT cells in the context of liver cirrhosis stimulate an increase in hepatic fibroblasts followed by increased secretion of IL-6 and IL-8, leading to profibrotic and proinflammatory effects. MAIT cells in NAFLD induce polarization of M2 macrophages, secrete more IL-4 and IL-10 and less IFN-γ, and play a protective role. Increased secretion of TNF-α, IFN-γ, IL-17, and IL-9 by MAIT cells exacerbates immunopathology in *Helicobacter pylori*–infected gastritis. In IBD, MAIT cells secrete amounts of TNF-α and IFN-γ, which play a pathogenic role. In PBC, increased secretion of proinflammatory cytokines by MAIT cells leads to enhanced inflammation. In viral hepatitis, MAIT cell production of the antiviral cytokine IFN-γ is increased and coculture of hepatocytes infected by HCV with MAIT cells limits viral replication (no researchers have studied the role of MAIT cells in HCV and PBC in animal models). ↑increase; ↓decrease.

Cancer of the colon and rectal area, which is collectively known as colorectal cancer, is the third most common cancer in the world.^60^ Won et al found that patients with colon cancer had significantly fewer MAIT cells in peripheral blood, whereas tumor tissue had more MAIT cells than adjacent normal tissue.^61^, ^62^, ^63^, ^64^ Circulating MAIT cells exhibited high levels of CCR6 and CXCR6, suggesting that the high frequency of MAIT cells in tumor tissues was associated with the migration of peripheral blood MAIT cells into colon cancer tissues.^64^ When MAIT cells were cocultured with HCT116 cells (human colorectal carcinoma cell line) in vitro, activated MAIT cells produced IFN-γ, TNF-α, and IL-17 and reduced HCT116 cell viability in a cell contact and MR1-dependent manner,^63^^,^^64^ thus suggesting a possible antitumor role of MAIT cells. However, another study found that increased tumor-infiltrating MAIT cells were associated with poor clinical outcome in colorectal cancer.^62^ Moreover, other researchers observed that MAIT cells produced IL-13 that enhanced colon tumor growth and metastasis via the STAT6 pathway,^65^ whereas the antitumor cytokine IFN-γ was reduced,^61^ possibly resulting in a protumor effect. Colorectal cancer is known to metastasize frequently to the liver. Shaler et al^66^ found that in patients with colorectal liver metastases, although MAIT cells infiltrated colorectal liver metastases, the number of MAIT cells in liver metastases was lower than in healthy liver tissue. Both tumor and tumor margin MAIT cells produced less IFN-γ than healthy liver tissue, suggesting that the tumor microenvironment contributes to MAIT cell dysfunction (Figure 2).^66^

## Mucosal-Associated Invariant T Cells in Hepatobiliary Diseases

### Autoimmune Liver Disease

The most common autoimmune liver diseases are autoimmune hepatitis, primary biliary cholangitis (PBC), and primary sclerosing cholangitis.^67^ Intrahepatic MAIT cells are mainly located around the bile ducts in the portal vein.^68^ In patients with autoimmune hepatitis, the MAIT cell frequency in peripheral blood cells was found to be reduced. Moreover, MAIT cell frequency in peripheral blood was negatively correlated with the stage of fibrosis and did not return to normal after immunosuppressive therapy.^69^^,^^70^ According to Jiang et al,^71^ a decrease in peripheral blood MAIT cells along with an increase in MAIT cells in the liver may be associated with an increase in the expression of CD25, activated caspase 3, integrin VLA-4, CXCR6, and CCR6 in peripheral blood in PBC.^68^^,^^72^ Increased IL-7 expression in hepatocytes and peripheral blood MAIT cells from patients with PBC and IL-7-induced STAT5 phosphorylation enhanced IFN-γ and TNF-α secretion in MAIT cells after TCR stimulation, which may further increase inflammation.^71^^,^^72^ In contrast, Setsu et al^73^ observed a significant reduction of MAIT cells in both blood and liver of patients with PBC perhaps because of the different stages of PBC. The same authors observed that the loss of peripheral MAIT cells persisted in patients who recovered normal levels of serum alanine aminotransferase, alkaline phosphatase, and γ-glutamyl transpeptidase levels after 6 months of treatment with ursodeoxycholic acid.^73^ In primary sclerosing cholangitis, patients had lower MAIT cells in peripheral blood and these cells expressed lower levels of CD107a, TNF-α, IFN-γ, and CD69 in MR1-dependent manner than healthy control subjects.^46^ In contrast, MAIT cells were significantly increased in bile duct tissue, which may be related to the presence of MAIT cell ligands in bile in patients with primary sclerosing cholangitis.^46^^,^^74^

### Viral Hepatitis

Infection with the hepatitis virus causes viral hepatitis, which can be acute or chronic in nature.^75^ According to Du et al,^76^ patients with acute HCV infection had a lower MAIT cell frequency in peripheral blood and reduced TNF-α and IFN-γ in response to *E coli* or IL-12 and IL-18 stimulation compared with healthy control subjects. Peripheral blood and intrahepatic MAIT cells were found to be reduced in patients with chronic hepatitis B, C, and D virus infection; however, residual peripheral blood MAIT cells were significantly activated.^77^, ^78^, ^79^, ^80^, ^81^, ^82^, ^83^, ^84^, ^85^ The virus cleared rapidly after antiviral treatment, but MAIT cell frequency, phenotype, and function remained comparable, suggesting irreversible changes.^8^^,^^79^^,^^82^ As compared with healthy control subjects, MAIT cells from CHBV-infected individuals secreted more IFN-γ, TNF-α, and IL-17A in response to TCR stimulation.^83^ In an in vitro assay, Liu et al^83^ reported that MAIT cells exhibited cytotoxicity against HBV-transfected hepatocytes in MR1-dependent manner, and Wilgenburg et al^44^ found that MAIT cells incubated with HCV-exposed macrophages restricted HCV replication in an IFN-γ-dependent manner. Altogether, these data suggest that MAIT cells may play an antiviral role in chronic viral hepatitis. As reported by Sandler et al,^86^ viral hepatitis infection and gut microbial translocation may be associated, which leads us to speculate whether MAIT cell activation can be partly attributed to gut bacteria.

### Alcohol-Related Liver Disease

Alcohol-related liver disease (ALD) is a type of liver disease caused by chronic heavy alcohol consumption. It usually manifests initially as fatty liver disease and can progress to alcoholic hepatitis, alcohol-related cirrhosis, and even liver cancer.^87^ Excessive alcohol consumption can affect the composition and abundance of the intestinal microbiota and is associated with increased transfer of microbial products from the gut to the liver via the portal circulation.^88^ Previously, Riva et al^89^ reported that peripheral blood MAIT cell frequency was significantly lower in patients with ALD (alcohol-related cirrhosis and severe alcoholic hepatitis) than in healthy volunteers, especially in patients with severe alcoholic hepatitis, where the decrease was most obvious. According to Zhang et al,^90^ the combined effects of IL-12, IL-18, and IL-8 could be responsible for the decrease in MAIT cells in patients with ALD. Compared with control subjects, peripheral blood MAIT cells expressed higher levels of CD69 and HLA-DR, but produced significantly less IL-17, granzyme B, and CD107a. Similar results were observed in in vitro experiments in which healthy peripheral blood mononuclear cells were exposed to fecal extracts of patients with ALD.^89^^,^^91^ Similarly, Gu et al^92^ reported reduced numbers of MAIT cells in the liver, intestine, and lungs of alcohol-fed C57 mice, and in human peripheral blood mononuclear cells exposed to alcohol-fed mouse serum or cecal microbiota in vitro. Therefore, it seems possible that excessive activation of MAIT cells by intestinal bacterial antigens and metabolites may contribute to their apoptosis and impair their antimicrobial function and cytotoxic response.^89^ Hegde et al^47^ found that long-term prophylactic use of antibiotics in patients with severe alcoholic cirrhosis partially prevented the reduction and activation of MAIT cells in the blood. Of note, Li et al^91^ found that the number of peripheral MAIT cells was not restored in patients with alcoholic hepatitis after 12 months of abstention from alcohol, suggesting that alcohol causes irreversible loss of MAIT cells.

### Nonalcoholic Fatty Liver Disease

Globally, nonalcoholic fatty liver disease (NAFLD) is the most prevalent chronic liver disease, and greater than 20% of the world's population suffers from NAFLD.^93^^,^^94^ Patients with NAFLD were found to have fewer circulating MAIT cells and a higher number of liver MAIT cells compared with healthy control subjects. Peripheral blood MAIT cells from patients with NAFLD were found to secrete higher levels of IL-4 and lower levels of IFN-γ and TNF-α. Moreover, in vitro activation of MAIT cells obtained from the peripheral blood of patients with NAFLD induced the polarization of M2 macrophages. Steatosis was more intense in MR1^-^/^-^ mice fed a methionine and choline deficient diet, supporting the hypothesis of a protective role of MAIT cells in NAFLD.^95^ Patients with NAFLD are often obese and suffer from diabetes, hypertriglyceridemia, and hypertension. Previous studies have reported that MAIT cells exacerbate inflammation in the ileum and adipose tissue in obese mice, inducing alterations in the gut microbiota and gut dysbiosis.^96^ However, in Asia, there are many patients with NAFLD with a normal body mass index,^97^ indicating that the effects of MAIT cells in NAFLD should be compared depending on the body mass index. However, dysbiosis of the intestinal flora in patients with NAFLD has been reported independently of the body mass index.^98^, ^99^, ^100^ The impact of the gut microbiota on MAIT cell phenotype and function in NAFLD is therefore an important topic for research.

### Liver Cirrhosis

Liver cirrhosis is the most severe form of liver fibrosis and is caused by a variety of factors, including HCV, ALD, and NAFLD.^101^^,^^102^ Niehaus et al^103^ reported that patients with decompensated cirrhosis had a lower frequency of peripheral blood MAIT cells and intrahepatic MAIT cells than healthy control subjects, and Vα7.2 cells were detected in the fibrotic septa accumulated in the liver. Moreover, in vitro activation of the MR1-dependent pathway in MAIT cells promoted the accumulation of hepatic myofibroblasts and stimulated their secretion of IL-6 and IL-8 via the production of TNF. Moreover, in the carbon tetrachloride (CCl4)-induced liver injury model, Vα19TCRTg mice showed enhanced fibrosis, whereas MR1^-^/^-^ mice showed reduced fibroblast numbers, indicating a profibrotic effect of MAIT cells.^47^ In decompensated cirrhosis with spontaneous bacterial peritonitis, MAIT cells were enriched in ascites and secreted more IFN-γ, TNF, and granzyme B in response to bacterial and cytokine stimulation compared with control subjects.^82^^,^^103^ Therefore, modulating the number and function of MAIT cells to fight infection may represent a therapeutic strategy that could be considered in the future.

### Cholangiocarcinoma

Cholangiocarcinoma is a malignant tumor of the intrahepatic or extrahepatic bile ducts.^104^^,^^105^ Zimmer et al^106^ found that MAIT cell frequency was reduced in tumor tissue compared with nontumor tissue in patients with cholangiocarcinoma, but tumor-infiltrating MAIT cells expressed high levels of the tissue-resident markers CD69 and CD103. The presence of tumor MAIT cells was positively correlated with survival and the signature of multiple innate immune cells, suggesting that MAIT cells may participate in antitumor immunity.^106^

### Hepatocellular Carcinoma

Hepatocellular carcinoma (HCC) is the main histologic subtype of liver cancer and is more prevalent in men compared with women.^107^ It has been reported that patients with HCC had fewer MAIT cells in peripheral blood and tumor tissue than healthy control subjects, but that the former cells displayed high expression of CD69, HLA-DR, and PD-1.^108^^,^^109^ Shaler et al^66^ also observed that HCC-infiltrated MAIT cells secreted significantly less IFN-γ, IL-17, granzyme B, and perforin, but more IL-8, and that high levels of HCC-infiltrating MAIT cells were associated with poor prognostic. These authors therefore hypothesized that MAIT cells might have protumor potential.^108^ However, Huang et al^109^ demonstrated that MAIT cells can display anticancer properties in vivo in mouse experiments. Thus, they observed that simultaneous stimulation by the synthetic riboflavin synthesis pathway–derived ligand 5-(2-oxopropylideneamino)-6-D-ribitylaminouracil (5-OP-RU) and the Toll-like receptor 9 agonist CpG reduced tumor burden and prolonged survival in a mouse model of HCC, and that protection was absent in MR1^-^/^-^ mice.^110^

## Conclusions and Future Directions

To summarize, MAIT cells are an important lymphocyte subset in the digestive system. MAIT cell development is strongly dependent on the symbiotic microbiota, but not all bacteria can activate MAIT cells. Many diseases, such as viral hepatitis, ALD, and NAFLD, are associated with changes in the composition and abundance of intestinal flora and with increased intestinal permeability, which can result in enhanced translocation of bacteria into the circulation or liver. Here, MAIT cells can be activated via the MR1 pathway or cytokine pathway, impacting their frequency, phenotype, and function in peripheral blood or tissues, and subsequently modulating disease activity. Yet, the interconnection among intestinal bacteria, MAIT cells, and disease needs further studies. MAIT cells are almost always found to be reduced in peripheral blood during digestive diseases, and the degree of peripheral blood MAIT cell depletion and activation correlates with disease severity.^57^^,^^72^^,^^83^^,^^89^^,^^95^ Moreover, peripheral MAIT cells undergo irreversible loss in viral hepatitis, PBC, and ALD. The loss of MAIT cells has been attributed to 2 causes: migration from the peripheral blood into tissues and overactivation leading to their death. Some authors argue that MAIT cells perform a proinflammatory or anti-inflammatory role by secreting certain cytokines, such as the proinflammatory cytokines IFN-γ, IL-17, and TNF-α and the anti-inflammatory cytokine IL-4. However, IL-17 may play a protective role in some cases. For example, the administration of anti-IL-17 antibodies to patients with Crohn's can exacerbate the disease.^111^ Cytokines are also key mediators in cancer, with IFN-γ contributing to the antitumor response^112^^,^^113^ and IL-17 helping to promote tumors.^114^^,^^115^ Based on cytokine secretion alone, it is difficult to predict whether MAIT cells play protective or pathogenic roles in the disease. Some in vitro experiments have shown that MAIT cells can directly kill tumor cells, yet definitive demonstration that this mechanism may operate in vivo is still lacking. Importantly, the number and function of MAIT cells in the same disease were found to vary between studies. Such differences may be caused by different tissue sites, disease stages, or sample selection, stressing the need to match gender, age, and tissue sites for accurate comparison between pathologic and control samples (Table 1).

**Table 1:** Definition, Frequency, Phenotype, Function, and Proposed Role of MAIT Cells in Digestive System Tumors

Disease type (species)	Definition	Frequency	Phenotype	Function	Proposed role	Reference
GC (human)	CD3^+^TCRγδ^−^ Vα7.2^+^CD161^+^	↓in blood	ND	Granzyme B↓ in blood	ND	^53^
EAC (human)	CD3^+^Vα7.2^+^ CD161^+^	↓in blood ↑in tissue	NKG2A↑PD-1↑/NKG2D↓ in tissue compared with blood	IFN-γ↓ TNF-α↓ in EAC TCM	Antitumor (OE33cell viability↓)	^54^
CRC (human)	CD3^+^TCRγδ^−^ Vα7.2^+^CD161^+^	↓in blood ↑in tissue	CD45RO^+^IL-18Rα^+^CD8^+^↓ in blood	TNF-α↓IFN-γ↓/IL-17A↑in blood IFN-γ↑IL-17↑ in tissue	Antitumor (HCT116cell viability↓)	^63^
CRC (human)	CD3^+^CD4^−^ TCRγδ−CD161^hi^ Vα7.2^+^	No difference in blood ↑in tissue	CD45RO/ CD69↓ in tissue	IFN-γ↓/IL-17↑ in tissue	Protumor (MAIT cell↑ = poor prognosis)	^61^^,^^62^
CRLM (human)	CD3^+^Vα7.2^+^CD161^+^ or CD3^+^MR1-tet^+^	↓in blood and tissue	ND	IFN-γ↓ granzyme B↓ in tissue	Tumor impairs MAIT cell function	^66^
CCA (human)	CD3^+^Vα7.2^hi^ MR1-tet^+^	↓in tissue	CD69↑CD103↑/CD25↓ HLA-DR↓CD57↓ in tissue	Perforin↓granzyme B↓ in tissue	Antitumor (MAIT cell↑ = good prognosis)	^106^
HCC (human)	CD3^+^Vα7.2^+^ CD161^+^MR1-tet^+^	↓in blood and tissue	PD-1↑CD160↓ in blood CCR7^−^CD45RA^−^ CD45RO^+^CD95^+^/CD160↓KLRG1↓CCR2↓ CD127↓CD28↓/CD38↑HLA-DR↑PD-1↑CTLA-4↑TIM-3↑CCR6↑CXCR6↑CCR9↑ in tissue	IFN-γ↓IL-17↓ in blood IFN-γ↓IL-17↓perforin↓ granzyme B↓/IL-8↑ in tissue	Protumor (MAIT cell↑ = poor prognosis)	^108^
HCC (5-OP-RU+ CpG in mice)	CD3^+^TCRβ^int^ MR1-tet^+^	ND	CD44^+^CD62L^–^/CD69↑ in tissue	IL-17A↓/IFN-γ↑ perforin↑granzyme B↑ in tissue	Antitumor (MAIT cell↑ = good prognosis)	^110^

NOTE. The ↑ symbol indicates an increase in frequency or expression levels, the ↓ symbol indicates a decrease in frequency or expression levels (compared with blood or tissue from healthy individuals or with unaffected tissues).

5-OP-RU, 5-(2-oxopropylideneamino)-6-D-ribitylaminouracil; CCA, cholangiocarcinoma; CCR, CC-chemokine receptor; CD45RO, memory T cell; CpG, Toll-like receptor 9 (TLR9) agonist; CRLM, colorectal liver metastases; CTLA-4, cytotoxic T-lymphocyte-associated protein 4; CXCR, CXC-chemokine receptor; EAC, esophageal adenocarcinoma; GC, gastric cancer; HCC, hepatocellular carcinoma; HCC (5-OP-RU + CpG), coadministration of 5-OP-RU and CpG in HCC mice; HLA-DR, human leukocyte antigen DR; IFN, interferon; IL, interleukin; In tissue, tumor tissue (CLRM tissue refers to liver metastasis tissue); MR1-tet^+^, MR1-5-OP-RU tetramer; NKG2A, also known as CD159, inhibitory receptor; NKG2D, also known as CD314, costimulatory receptor; ND, not determined; PD-1, programmed cell death 1; TCM, tumor conditioned medium; TIM-3, T cell immunoglobulin and mucin 3; TNF, tumor necrosis factor.

Further studies notably combining in vitro and in vivo approaches remain necessary to ascertain their precise roles in different diseases. Such knowledge will be indispensable to determine whether and how MAIT cell agonists or MR1 blockade may become a therapeutic approach in some diseases. Recent work in respiratory diseases, rheumatic diseases, and endocrine diseases similarly revealed versatile effects of MAIT cells, raising questions on their respective effects and suggesting that the role of MAIT cells may largely vary depending on disease and context in the tissue.^15^^,^^116^, ^117^, ^118^ Overall, the functions of MAIT cells in various diseases remain largely elusive and more work is needed to define the molecular mechanisms that underlie their variable functions.
